# Inequalities in early childhood mortality in Myanmar - *Association between parents’ socioeconomic status and early childhood mortality*

**DOI:** 10.1080/16549716.2019.1603516

**Published:** 2019-05-08

**Authors:** Sai San Moon Lu, Jennifer Stewart Williams, Johan Nilsson Sommar

**Affiliations:** a Department of Preventive and Social Medicine, University of Medicine Mandalay, Mandalay, Myanmar; b Department of Epidemiology and Global Health, Umeå University, Umeå, Sweden; c Research Centre for Generational Health and Ageing, Faculty of Health, University of Newcastle, New Lambton Heights, NSW, Australia; d Division of Occupational and Environmental Medicine, Department of Public Health and Clinical Medicine, Umeå University, Umeå, Sweden

**Keywords:** Household wealth, parental education, neonatal mortality, post-neonatal mortality, under-5 mortality

## Abstract

**Background**: Despite global achievements in reducing early childhood mortality, disparities remain. There have been empirical studies of inequalities conducted in low- and middle-income countries. However, there have been no epidemiological studies on socioeconomic inequalities and early childhood survival in Myanmar.

**Objective**: To estimate associations between two measures of parental socioeconomic status – household wealth and education – and age-specific early childhood mortality in Myanmar.

**Methods**: Using cross-sectional data obtained from the Myanmar Demographic Health Survey (2015–2016), univariate and multiple logistic regressions were performed to investigate associations between household wealth and highest attained parental education, and under-5, neonatal, post-neonatal and child mortality. Data for 10,081 children born to 5,932 married women (aged 15–49 years) 10 years prior to the survey, were analysed.

**Results**: Mortality during the first five years was associated with household wealth. In multiple logistic models, wealth was protective for post-neonatal mortality. After adjusting for individual proximate determinants, the odds of post-neonatal mortality in the richest households were 85% lower (95% CI: 50–96%) than in the poorest households. However, significant association was not found between wealth and neonatal mortality. Parental education was important for early childhood mortality; the highest benefit from parental education was for child mortality in the one- to five-year age bracket. After adjusting for proximate determinants, children with a higher educated parent had 95% (95% CI 77–99%) lower odds of death in this age group compared with children whose parents’ highest educational attainment was at primary level. The association between parental education and neonatal mortality was not significant.

**Conclusions**: In Myanmar, household wealth and parental education are important for childhood survival before five years of age. This study identified nuanced age-related differences in associations. Health policy must take socioeconomic determinants into account in order to address unfair inequalities in early childhood mortality.

## Background

Global trends in childhood mortality show major improvements in the under-5 age group [1–3]. The Global Burden of Disease (GBD) Study reported a 33% decrease in under-5 deaths (2006–2016) from 7.46 million to 5.00 million, with neonatal mortality (birth to 28 days) accounting for 43.3% of all under-5 deaths in 2016. For the same period, the drop in neonatal mortality was 28.9% [4]. According to the 2017 United Nations (UN) Child Mortality Report, the global under-5 mortality rate declined from 93 deaths per 1,000 live births in 1990 to 41 deaths per 1,000 live births in 2016 – a drop of 56% [5].

However, disparities in childhood mortality still exist within and across regions and countries [5]. Children in Sub-Saharan African (SSA) countries have a 20 times higher risk of dying before the age of five, compared to children living in the Australasian Region (Australia and New Zealand), and more than 90% of deaths in children under-5 occur in low- and middle-income countries (LMICs) [5,6]. A systematic analysis by the GBD Collaboration showed that between 1970 and 2016, neonatal mortality in countries classified as having a low socio-demographic index (SDI) was 24.3 deaths per 1,000 live births compared with 2.7 deaths per 1,000 live births in high SDI countries. The comparable rates for post-neonatal mortality (one month to one year) in low versus high SDI countries, were 23.2 and 1.4 deaths per 1,000 live births, and among children aged between one and five years, 24.4 and 0.8 deaths per 1,000 live births, respectively [2]. A study which systematically compared household wealth inequalities in child mortality in 10 African cities between 2000, 2007 and 2011 showed wide disparities across time and place [7].

In Myanmar the GBD estimate of under-5 deaths per 1,000 live births (1970–2016) was 27.7 compared with 22.3 in the Southeast Asia Region. The largest share was in the neonatal period being 15.0 per 1,000 live births [2]. In the same period, under-5 mortality was 6.4 deaths per 1,000 live births in neighbouring Thailand, and 13.1 per 1,000 live births in Vietnam [2]. Neonatal deaths were also lower in Thailand and Vietnam compared with Myanmar, being 3.3 and 7.0 per 1,000 live births, respectively [2].

Socioeconomic inequalities in childhood mortality are an important public health issue in many LMICs [5]. In India the 2015 U5MR was estimated at 28 deaths per 1,000 live births in urban areas, compared with 48 deaths per 1,000 live births in rural areas [5,8]. The UN Inter-agency Group for Child Mortality Estimation (UN IGME) reported that, out of 99 surveyed LMICs, children born to the poorest families were on average, twice as likely to die before the age of five compared with children born to the wealthiest families [5]. Using data from 52 LMICs in the Demographic Health Surveys (DHSs) between 2000 and 2011, Van Deurzen et al. found that more women experienced child mortality in countries with higher wealth inequality [9]. In another study which used DHS data (2002–2012) from LMICs, Bendavid showed that under-5 mortality was falling faster among the poorest families compared with the least poor [10]. National income is also an important determinant of child survival. The results of a pooled meta-analysis showed that larger increases in Gross Domestic Product per Purchasing Power Parity were needed to reduce child mortality in the poorer countries [11]. Reducing early childhood mortality across all wealth groups in all countries is important for global population health [12].

Although global childhood mortality has declined, it is important to continue to achieve progress [1]. More work is needed in order to meet the UN Sustainable Development Goal (SDG) targets of below 25 deaths per 1,000 live births in under-5 mortality by 2030 [2,13]. Even if all countries meet these SDG targets, it is estimated that there will still be 56.0 million under-5 deaths by 2030. The regions with the highest under-5 mortality rates are SSA and South Asia. Yet in formulating policies to reduce childhood mortality it is important to understand how socioeconomic factors, for example parental education and household wealth, impact on early childhood survival [14].

Since the association between mothers’ education and childhood mortality in Nigeria was investigated by Caldwell in 1979 [15], there has been further interest in this area [12]. An analysis of DHS data in seven SSA countries showed that children of mothers who did not attend school had a higher rate of death compared with children whose mothers had formal education [16], and a recent study across 43 LMICs suggests a weakening association between parental education and child health outcomes [17].

Myanmar – formerly known as Burma – is a low-middle-income country [18] in Southeast Asia with a population of 54 million [19]. The 2017 World Bank Poverty Report showed that, based on the Gini coefficient, inequality in Myanmar was below that of other countries in the Region [20]. However almost one-third of the population lived below the poverty line in 2015 [20] and half of the rural population (51%) was classified as belonging to the lowest (poorest) and second lowest household wealth quintiles, compared with only 9% of the urban population [21]. Thirty-four percent of women and 29% of men from the poorest wealth quintiles had never attended formal schools, compared with only 10% of women and 9% of men from the wealthiest households [21].

Consistent with the global trend, there have been remarkable reductions in early childhood mortality in Myanmar in the past decade. Between 2001–2002 and 2010–2011 neonatal mortality fell from 38 deaths per 1,000 live births to 25 deaths per 1,000 live births, post-neonatal mortality fell from 46 deaths per 1,000 live births to 16 deaths per 1,000 live births. In 2012 under-5 mortality in Myanmar decreased from 103 deaths per 1,000 live births to 50 deaths per 1,000 live births [21]. However according to the 2015–16 Myanmar DHS [21] the under-5 mortality rate ranged from 26 to 99 deaths per 1,000 live births between the richest and poorest households. In addition to data reported in official documents, rigorous epidemiological studies are needed to provide an evidence base for policy in Myanmar.

This study therefore aims to fill an evidence gap by estimating the association between two measures of parental socioeconomic status – household wealth and parental education – and age-specific early childhood mortality in Myanmar. Although there is considerable evidence of global trends and inequalities in child mortality, epidemiological studies that use representative national data to better understand and monitor inequalities within countries, are needed [4,22–24].

## Methods

### Study data and conceptual framework

Cross-sectional data obtained from the 2015–16 Myanmar Demographic Health Survey data (MDHS) were used in this study [21]. A nationally representative sample of 12,885 women and 4,737 men age 15–49 in 12,500 selected households were interviewed. Response rates were 96% and 91% for women and men, respectively.

Information on child mortality was collected as a part of retrospective birth histories in which women (aged 15–49 years) were asked to list all children they had borne, and each child’s date of birth, survival status, and current age or age at death [21]. For this study, birth history data were collected from 5,932 women (aged 15–49 years) who delivered 10,081 live births within 10 years prior to the survey.

**Figure 1.**
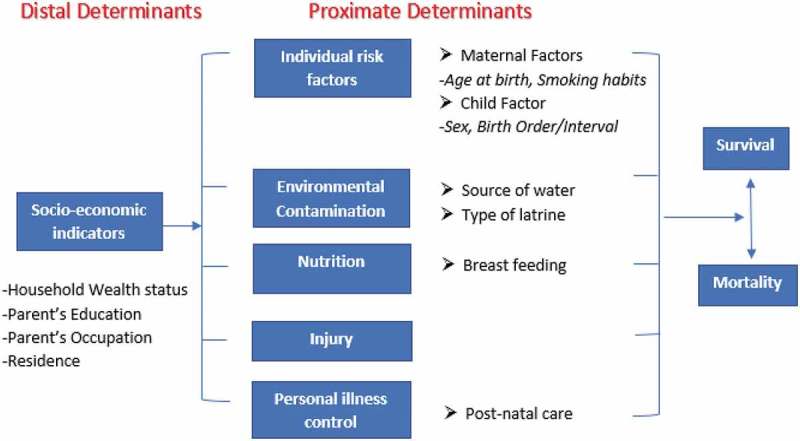
Conceptual framework showing distal and proximate determinants of childhood mortality adopted from Mosley and Chen.

The theoretical basis for the analysis was derived from the Framework proposed by Mosley and Chen for the purpose of studying child survival in LMICs [25]. This is based on the premise that far-reaching socioeconomic determinants such as household wealth and parental education influence child survival through individual risk factors and other immediate proximate determinants. All variables in the analyses were derived from the 2015–16 MDHS dataset and the Mosley and Chen Framework was used for variable selection. See Figure 1 for details.

### Dependent variables

The dependent variables were defined in accordance with established early childhood mortality age cut-offs [26,27] as: ‘under-5 mortality’ (deaths between birth and five years of age); ‘neonatal mortality’ (deaths occurring in the first 28 days of life); ‘post-neonatal mortality’ (deaths between one month and one year of age), and ‘child mortality’ (deaths between one and five years of age).

### Exposure variables

The exposure variables are household wealth and (highest attained) parental education, both of which are indicators of socioeconomic status and are classified as distal determinants within the Mosley and Chen Framework [25]. Household wealth was measured by an index based on household ownership of selected consumer goods such as a television, bicycle or car, housing characteristics such as flooring, source of drinking water and sanitation facilities [21]. Scores were derived using principal component analysis with results grouped into five wealth quintiles referred to here as the poorest, poorer, middle, richer and richest. Parental education was measured as the highest self-reported educational level attained by either parent. Only two-parent families were included in these analyses. Education was categorised as no education, primary school, secondary school and post-secondary school.

### Covariates

Three groups of variables – 1) distal socioeconomic, 2) individual proximate, and 3) nutrition and personal illness control – were included in the analyses. In addition to the exposure variables, the distal socioeconomic determinants were: mother’s occupation, father’s occupation (both categorised as professional/technical/managerial, sales and services, agriculture, unskilled manual and not working) and residency (urban or rural). Individual proximate determinants were: age of mother at child birth (< 20, 20–29, 30–39 and ≥ 40 years), preceding birth interval (‘≤ 24 months’, ‘> 24 months interval or 1st birth order’), mothers’ use of cigarettes or tobacco (‘use’ or ‘no use’), sex of the children (male or female) and birth order (1, 2–3, 4–6 and ≥ 7). The nutrition determinant was breastfed (never breastfed or ever breastfed) and the determinant indicating personal illness control was postnatal care received within two months after the birth (categorised as ‘yes’, ‘no’ and ‘don’t know’).

### Statistical analysis

Descriptive statistics are presented to show numbers and proportions of live births and deaths by early childhood mortality groups. Concentration curves provide a visual presentation of under-5 mortality by household wealth and parental education.

Univariate logistic regression (Model A) was used to separately investigate the crude association between each of the exposure variables (household wealth and parental education) and each of the four age-specific mortality outcomes (under-5 mortality, neonatal mortality, post-neonatal mortality, and child mortality). Multiple logistic regression additionally adjusted for individual proximate determinants (Model B). Thereafter distal variables were added to these multiple logistic models (Model C), and in the last step nutrition and personal illness control variables (Models D and E).

The proportion of missing values in nutrition and personal illness control exceeded 20% and these variables were therefore not included in the main models. Sensitivity analyses (Models D and E) were performed on a reduced dataset that included only records with non-missing observations for the nutrition and personal illness control variables. The purpose was to assess the robustness of the main results in Model B.

For the regressions the reference category for household wealth was the poorest wealth quintile and for parental education the reference category was primary school. Associations were reported as odds ratios with 95% confidence intervals. Survey sampling weights were used in all analyses. The Hosmer-Lemeshow Goodness-of-Fit test was performed on all models. All analyses were undertaken using Stata version 12 (StataCorp).

## Results

### Characteristics of the children and parents

There were 10,081 live births in total within the 10 years prior to the 2015–2016 MDHS and among these, 650 children died before five years of age. (See Table 1).

**Table 1.** Numbers and proportions of live births and deaths in the under-5, neonatal, post-neonatal and child periods by socioeconomic characteristics.

Socioeconomic characteristics	Under-5 period^1^		Neonatal period^2^		Post-neonatal period^3^		Child period^4^	
	N (%)	Deaths (%)	N (%)	Deaths (%)	N (%)	Deaths (%)	N (%)	Deaths (%)
**Household Wealth Index**	10,081 (100)	650 (100)	10,081 (100)	321 (100)	9760 (100)	237 (100)	9523 (100)	92 (100)
*Richest*	1213 (12)	32 (5)	1213 (12)	23 (7)	1190 (12)	6 (3)	1184 (12)	3 (3)
*Richer*	1601 (16)	56 (9)	1601 (16)	32 (10)	1569 (16)	16 (7)	1553 (16)	8 (9)
*Middle*	1870 (19)	116 (18)	1870 (19)	62 (19)	1808 (19)	39 (16)	1769 (19)	15 (16)
*Poor*	2378 (24)	184 (28)	2378 (24)	100 (31)	2278 (23)	64 (27)	2214 (23)	20 (22)
*Poorest*	3019 (30)	262 (40)	3019 (30)	104 (32)	2915 (30)	112 (47)	2803 (29)	46 (50)
**Mother’s Education**	10,081 (100)	650 (100)	10,081 (100)	321 (100)	9760 (100)	237 (100)	9523 (100)	92 (100)
*Higher*	596 (6)	15 (2)	596 (6)	13 (4)	583 (6)	0 (0)	583 (6)	2 (2)
*Secondary*	2779 (28)	126 (19)	2779 (28)	78 (24)	2701 (28)	32 (14)	2669 (28)	16 (17)
*Primary*	4715 (47)	319 (49)	4715 (47)	154 (48)	4561 (47)	124 (52)	4437 (47)	41 (45)
*No education*	1990 (20)	190 (29)	1990 (20)	76 (24)	1914 (20)	81 (34)	1833 (19)	33 (36)
*Missing*	1 (0)	0 (0)	1 (0)	0 (0)	1 (0)	0 (0)	1 (0)	0 (0)
**Father’s Education**	10,081 (100)	650 (100)	10,081 (100)	321 (100)	9760 (100)	237 (100)	9523 (100)	92 (100)
*Higher*	472 (5)	14 (2)	472 (5)	13 (4)	459 (5)	0 (0)	459 (5)	1 (1)
*Secondary*	3451 (34)	174 (27)	3451 (34)	101 (31)	3350 (34)	52 (22)	3298 (35)	21 (23)
*Primary*	4103 (41)	280 (43)	4103 (41)	131 (41)	3972 (41)	108 (46)	3864 (41)	41 (45)
*No education*	1860 (18)	172 (26)	1860 (18)	71 (22)	1789 (18)	73 (31)	1716 (18)	28 (30)
*Missing/don’t know*	195 (2)	10 (2)	195 (2)	5 (2)	190 (2)	4 (2)	186 (2)	1 (1)
**Mother’s Occupation**	10,081 (100)	650 (100)	10,081 (100)	321 (100)	9760 (100)	237 (100)	9523 (100)	92 (100)
*Professional/technical*	414 (4)	15 (2)	414 (4)	9 (3)	405 (4)	2 (1)	403 (4)	4 (4)
*Sales/Services/Clerical*	1989 (20)	105 (16)	1989 (20)	61 (19)	1928 (20)	32 (14)	1896 (20)	12 (13)
*Agricultural*	1830 (18)	158 (24)	1830 (18)	71 (22)	1759 (18)	60 (25)	1699 (18)	27 (29)
*Unskilled*	2491 (25)	199 (31)	2491 (25)	87 (27)	2404 (25)	84 (35)	2320 (24)	28 (30)
*Not Working*	3336 (33)	171 (26)	3336 (33)	93 (29)	3243 (33)	58 (24)	3185 (33)	20 (22)
*Missing*	21 (0)	2 (0)	21 (0)	0 (0)	21 (0)	1 (0)	20 (0)	1 (1)
**Father’s Occupation**	10,081 (100)	650 (100)	10,081 (100)	321 (100)	9760 (100)	237 (100)	9523 (100)	92 (100)
*Professional/technical*	701 (7)	30 (5)	701 (7)	15 (5)	686 (7)	8 (3)	678 (7)	7 (8)
*Sales/Services/Clerical*	2421 (24)	111 (17)	2421 (24)	62 (19)	2359 (24)	41 (17)	2318 (24)	8 (9)
*Agricultural*	2912 (29)	211 (32)	2912 (29)	102 (32)	2810 (29)	73 (31)	2737 (29)	36 (39)
*Unskilled*	3954 (39)	290 (45)	3954 (39)	137 (43)	3817 (39)	113 (48)	3704 (39)	40 (43)
*Not Working*	2 (0)	0 (0)	2 (0)	0 (0)	2 (0)	0 (0)	2 (0)	0 (0)
*Missing*	91 (1)	8 (1)	91 (1)	5 (2)	86 (1)	2 (1)	84 (1)	1 (1)
**Type of Residence**	10,081 (100)	650 (100)	10,081 (100)	321 (100)	9760 (100)	237 (100)	9523 (100)	92 (100)
*Urban*	2119 (21)	74 (11)	2119 (21)	40 (12)	2079 (21)	28 (12)	2051 (22)	6 (7)
*Rural*	7962 (79)	576 (89)	7962 (79)	281 (88)	7681 (79)	209 (88)	7472 (78)	86 (93)

^1^between birth and five years of age, ^2^the first 28 days of life, ^3^between one month and one year of age, ^4^between one and five years of age.

**Figure 2.1.**
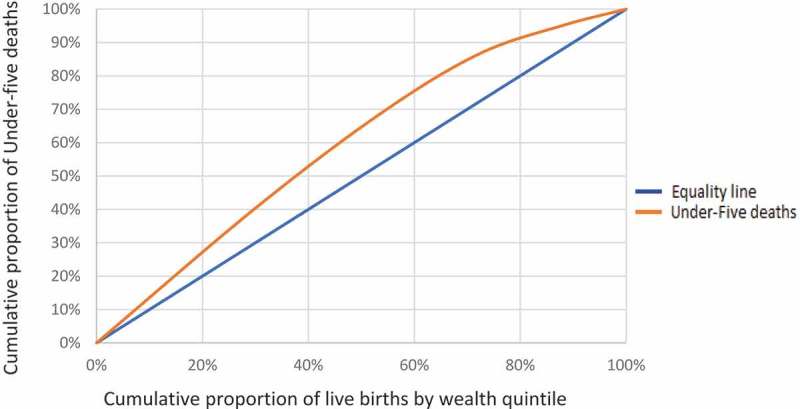
The concentration curve showing the distribution of under-5 deaths and numbers of live births ranked by household wealth.

Figure 2.1 plots the cumulative proportion of live births ranked by household wealth, against the cumulative proportion of under-5 deaths with a reference line of hypothetical equality. Out of 20% of live births in the relatively poorest families, under-5 mortality was over 25%, whereas for higher wealth families, under-5 mortality for the same proportion of live births was less than 10%.

**Figure 2.2.**
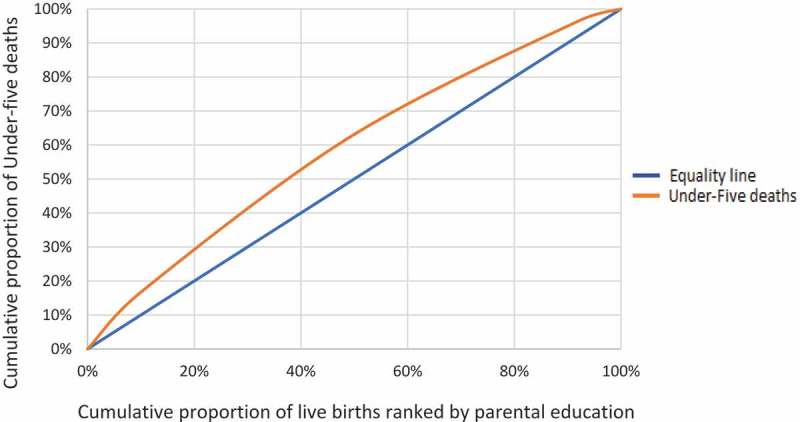
The concentration curve showing the distribution of under-5 deaths and numbers of live births ranked by highest attained parental education.

Similarly Figure 2.2 plots the cumulative proportion of live births ranked by parental education, against the cumulative proportion of under-5 deaths with a reference line of hypothetical equality. Of the 20% of live births in families with relatively low parental education, under-5 mortality was 30%. In families with the highest parental education, however, under-5 mortality in 20% of live births was about 12% (Figure 2.2).

### Association between household wealth status and under-5 mortality (*Figure 3.1*)

Children in the richest, richer and middle household wealth groups had significantly lower odds of dying before age five compared with children from the poorest households (Model A), suggesting a wealth gradient in under-5 mortality. After adjusting for the individual determinants in Model B, this wealth gradient remained significant among the richest and richer household wealth groups. Compared with the poorest families, children from the richest and rich wealth quintiles had 67% (95% CI 44–80%) and 49% (95% CI 16–69%) lower odds of dying before the age of five. Model B showed the best fit according to Akaike information criterion.

### Association between highest attained parental education and under-5 mortality (*Figure 3.2*)

Children with either secondary or post-secondary highest attained parental education had significantly lower odds of dying before age five compared with children with highest attained primary parental education (Model A). Compared with children whose parents had attended only primary school, children born to parents who had attended either secondary or post-secondary school had 43% (95% CI 27–56%) and 64% (95% CI 34–81%) lower odds of mortality, respectively. After adjusting for individual proximate determinants (Model B) children with secondary and post-secondary highest attained parental education had 34% (95% CI 15–49%) and 55% (95% CI 14–76%) lower odds of dying, respectively, compared with children whose parents’ highest attained education was primary. Model B showed the best fit also for parental education according to the Akaike information criterion.

### Association between household wealth status and age-stratified early childhood mortality (*Table 2.1*)

In the univariate analysis, the association between wealth and mortality was generally stronger during the post-neonatal and child periods compared to the neonatal period (Model A). Compared with the poorest families, the odds of post-neonatal death were 90% (95% CI: 66–97%), 73% (95% CI: 42–88%) and 47% (95% CI: 15–66%) lower among the richest, richer and middle wealth families, respectively (Model A). The odds of child mortality (i.e. between one and five years of age) were 76% (95% CI: 2–94%) and 61% (95% CI: 4–84%) lower among the richest and richer families, respectively. The wealth gradient persisted after the neonatal period in the presence of individual proximate determinants (Model B). Yet, the association with wealth remained significant only for richest and richer families during the post-neonatal period. The odds of post-neonatal mortality were 85% (95% CI: 50–96%) and 64% (95% CI: 20–83%) lower among children in the richest and richer families, respectively, compared with children in the poorest families (Model B).

**Table 2.1.** Associations between household wealth and age-stratified early childhood mortality (neonatal, post-neonatal and child mortality).

Household Wealth Index (ref: Poorest)		Neonatal mortality^1^ OR (95% CI)	Post-neonatal mortality^2^ OR (95% CI)	Child mortality^3^ OR (95% CI)
Univariate Analysis	**Model A**	**N = 10,081**	**N = 9760**	**N = 9523**
	*Richest*	0.47*[0.26–0.85]	0.10***[0.03–0.34]	0.24*[0.06–0.98]
	*Richer*	0.61[0.35–1.07]	0.27***[0.12–0.58]	0.39*[0.16–0.96]
	*Middle*	0.83[0.53–1.29]	0.53**[0.34–0.85]	0.68[0.34–1.38]
	*Poor*	1.30[0.84–2.02]	0.68[0.47–1.00]	0.80[0.40–1.60]
Multiple logistic models	**Model B**	**N = 10,081**	**N = 9760**	**N = 9523**
	*Richest*	0.54[0.28–1.03]	0.15**[0.04–0.50]	0.36[0.09–1.50]
	*Richer*	0.69[0.39–1.23]	0.36*[0.17–0.80]	0.53[0.20–1.40]
	*Middle*	0.91[0.58–1.42]	0.65[0.40–1.06]	0.83[0.39–1.74]
	*Poor*	1.38[0.89–2.16]	0.77[0.52–1.13]	0.90[0.43–1.85]
	**Model C**	**N = 9791**	**N = 8715**	**N = 9249**
	*Richest*	0.71[0.36–1.40]	0.23[0.05–1.04]	0.88[0.20–3.89]
	*Richer*	0.83[0.45–1.51]	0.45[0.18–1.12]	0.81[0.28–2.39]
	*Middle*	0.93[0.58–1.48]	0.74[0.45–1.21]	1.01[0.44–2.32]
	*Poor*	1.34[0.85–2.12]	0.85[0.57–1.26]	0.95[0.45–2.00]
Sensitivity analysis	**Model D**	**N = 3866**	**N = 3809**	**N = 3254**
	*Richest*	0.58[0.14–2.41]	0.32[0.04–2.32]	1[[1-]]
	*Richer*	0.77[0.25–2.39]	0.64[0.11–3.70]	0.33[0.07–1.64]
	*Middle*	0.69[0.27–1.80]	1.14[0.23–5.72]	0.22[0.03–1.99]
	*Poor*	1.35[0.58–3.14]	1.13[0.29–4.37]	0.41[0.08–2.06]
	**Model E**	**N = 3866**	**N = 3805**	**N = 3208**
	*Richest*	0.57[0.15–2.08]	0.35[0.05–2.43]	1[[1-]]
	*Richer*	0.60[0.20–1.79]	0.68[0.12–3.85]	0.32[0.06–1.54]
	*Middle*	0.66[0.25–1.78]	1.24[0.27–5.76]	0.23[0.03–2.05]
	*Poor*	1.31[0.48–3.56]	1.12[0.30–4.25]	0.43[0.08–2.23]

Note: Model A assessed the univariate association between household wealth index and each of the four mortality outcomes. Model B assessed the association between household wealth index and each of the four mortality outcomes considering also the individual proximate determinants (birth interval, birth order, mothers’ use of cigarettes/tobacco, maternal age at birth and sex of the child). Model C additionally adjusts Model B for the distal determinants (highest attained parental education, mothers’ occupation, fathers’ occupation and residence). Model D (sensitivity analysis) repeats Model B on a reduced data set that includes only records with non-missing values for the nutrition (breastfeeding) and personal illness control (postnatal care) variables. Model E additionally adjusts Model D for nutrition (breastfeeding) and personal illness control (postnatal care) variables.

* p < 0.05, ** p < 0.01, *** p < 0.001.

^1^deaths occurring in the first 28 days of life, ^2^deaths between one month and one year of age, ^3^deaths between one and five years of age.

### Association between highest attained parental education and age-stratified early childhood mortality (*Table 2.2*)

In the univariate analysis, parental education (Model A) was associated with mortality within all age groups, with the largest benefits observed for child mortality (i.e. between one and five years of age). After adjusting for the individual proximate determinants in Model B, significant association was evident only after the neonatal period. Compared with children whose highest attained parental education was primary, children with secondary parental education had 41% (95% CI: 6–63%) lower odds of dying during the post-neonatal period. The odds of child mortality were 95% (95% CI: 77–99%) lower among children with parents with post-secondary education compared with children whose parents’ highest attained education was primary. Model B showed the best fit.

**Table 2.2.** Associations between highest attained parental education level and age-stratified early childhood mortality (neonatal, post-neonatal and child mortality).

Highest attained parental education (ref: Primary school)		Neonatal mortality^1^ OR (95% CI)	Post-neonatal mortality^2^ OR (95% CI)	Child mortality^3^ OR (95% CI)
Univariate Analysis	**Model A**	**N = 9885**	**N = 8796**	**N = 9336**
	*Post-secondary*	0.80[0.42–1.52]	1[1.00–1.00]	0.04***[0.01–0.17]
	*Secondary school*	0.66*[0.48–0.91]	0.48**[0.31–0.76]	0.57[0.32–1.02]
	*no education*	1.03[0.68–1.56]	1.35[0.85–2.12]	2.36*[1.15–4.83]
Multiple logistic models	**Model B**	**N = 9885**	**N = 8796**	**N = 9336**
	*Post-secondary*	0.91[0.45–1.86]	1[1.00–1.00]	0.05***[0.01–0.23]
	*Secondary school*	0.72[0.51–1.00]	0.59*[0.37–0.94]	0.69[0.40–1.17]
	*no education*	0.91[0.58–1.43]	1.09[0.71–1.67]	1.99[0.94–4.24]
	**Model C**	**N = 9791**	**N = 8715**	**N = 9249**
	*Post-secondary*	1.70[0.79–3.66]	1[1.00–1.00]	0.08**[0.01–0.53]
	*Secondary school*	0.91[0.64–1.30]	0.74[0.45–1.22]	0.83[0.44–1.55]
	*no education*	0.96[0.60–1.52]	1.18[0.78–1.78]	2.04[0.93–4.46]
Sensitivity analysis	**Model D**	**N = 3787**	**N = 3337**	**N = 3307**
	*Post-secondary*	1.64[0.43–6.30]	1[1.00–1.00]	1[1.00–1.00]
	*Secondary school*	1.24[0.52–2.93]	0.94[0.21–4.12]	0.83[0.29–2.39]
	*no education*	1.29[0.42–3.95]	0.99[0.26–3.70]	1.05[0.15–7.26]
	**Model E**	**N = 3787**	**N = 3333**	**N = 3262**
	*Post-secondary*	1.88[0.52–6.77]	1[1.00–1.00]	1[1.00–1.00]
	*Secondary school*	1.18[0.50–2.80]	0.95[0.21–4.37]	0.81[0.28–2.32]
	*no education*	0.77[0.22–2.68]	0.97[0.26–3.60]	0.96[0.14–6.57]

Note: Model A assessed the association between highest attained parental education level and each of the four mortality outcomes. Model B assessed association between highest attained parental education level and each of the four mortality outcomes considering also the individual proximate determinants (birth interval, birth order, mothers’ use of cigarettes/tobacco, maternal age at birth and sex of the child). Model C additionally adjusts Model B for the distal determinants (household wealth index, mothers’ occupation, fathers’ occupation and residence). Model D (sensitivity analysis) repeats Model B on a reduced data set that includes only records with non-missing values for the nutrition (breastfeeding) and personal illness control (postnatal care) variables. Model E additionally adjusts Model D for nutrition (breastfeeding) and personal illness control (postnatal care) variables.

* p < 0.05, ** p < 0.01, *** p < 0.001.

^1^deaths occurring in the first 28 days of life, ^2^deaths between one month and one year of age, ^3^deaths between one and five years of age.

### Sensitivity analysis (models d and E)

**Figure 3.1.**
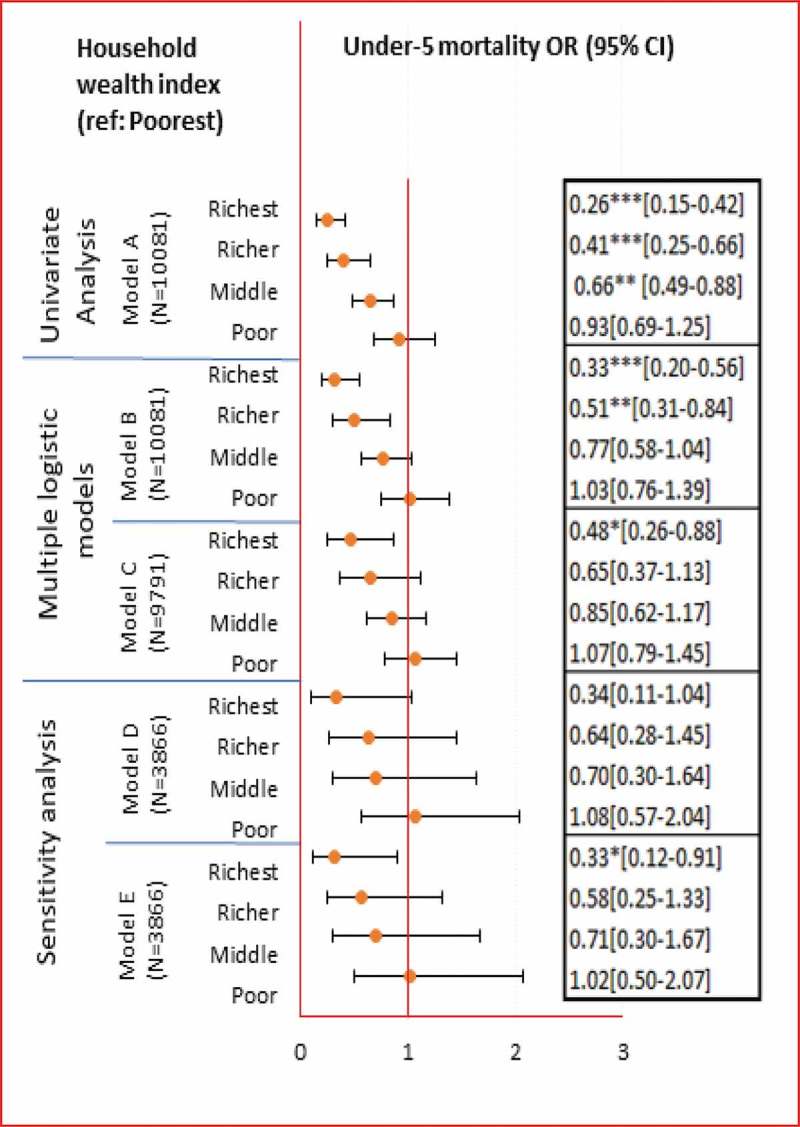
Associations between household wealth and under-5 mortality. Note: Model A assessed the univariate association between household wealth index and each of the four mortality outcomes. Model B assessed the association between household wealth index and each of the four mortality outcomes considering also the individual proximate determinants (birth interval, birth order, mothers’ use of cigarettes/tobacco, maternal age at birth and sex of the child). Model C additionally adjusts Model B for the distal determinants (highest attained parental education, mothers’ occupation, fathers’ occupation and residence). Model D (sensitivity analysis) repeats Model B on a reduced data set that includes only records with non-missing values for the nutrition (breastfeeding) and personal illness control (postnatal care) variables. Model E additionally adjusts Model D for nutrition (breastfeeding) and personal illness control (postnatal care) variables.* p < 0.05, ** p < 0.01, *** p < 0.001 Under-5 mortality: deaths between birth and five years of age.

**Figure 3.2.**
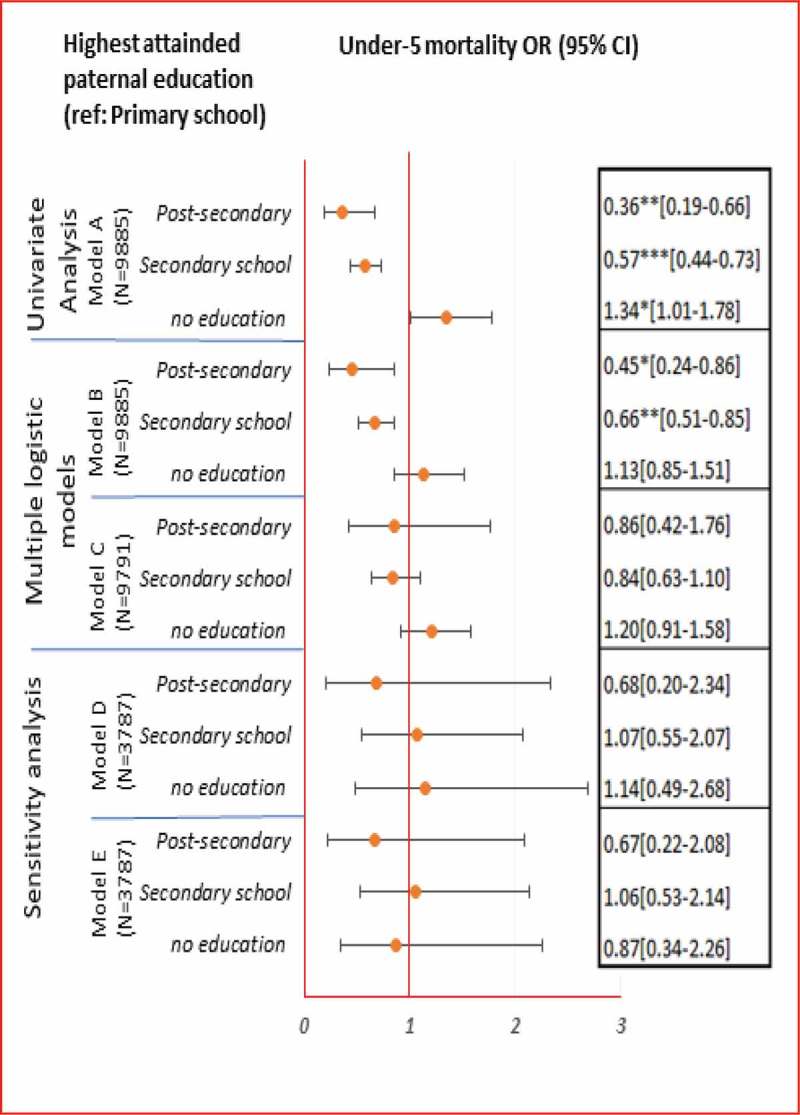
Associations between highest attained parental education and under-5 mortality. Note: Model A assessed the association between highest attained parental education level and each of the four mortality outcomes. Model B assessed association between highest attained parental education level and each of the four mortality outcomes considering also the individual proximate determinants (birth interval, birth order, mothers’ use of cigarettes/tobacco, maternal age at birth and sex of the child). Model C additionally adjusts Model B for the distal determinants (household wealth index, mothers’ occupation, fathers’ occupation and residence). Model D (sensitivity analysis) repeats Model B on a reduced data set that includes only records with non-missing values for the nutrition (breastfeeding) and personal illness control (postnatal care) variables. Model E additionally adjusts Model D for nutrition (breastfeeding) and personal illness control (postnatal care) variables.* p < 0.05, ** p < 0.01, *** p < 0.001 Under-5 mortality: deaths between birth and five years of age.

The sensitivity analysis aimed to assess the robustness of the main result presented in Model B by considering also nutrition and personal illness control variables. It is conceivable that breastfeeding and postnatal care were on the causal pathway and possible mediating effects were observed for household wealth and neonatal mortality (Figure 3.1, Table 2.1: Models D and E). In this case when the richer families were compared with the poorest, the odds of death in the neonatal period were 23% lower in Model D, but 40% lower in Model E (Table 2.1). Breastfeeding and postnatal care may have also mediated the association between parental education and neonatal death (Figure 3.2, Table 2.2: Models D and E); the odds of death in the neonatal period were 29% higher in Model D but 33% lower after adjusting for breastfeeding and postnatal care in Model E.

## Discussion

To the best of our knowledge this is the first study in Myanmar to empirically investigate parental socioeconomic status and early childhood mortality. The inverse association between household wealth and under-5 mortality suggested a wealth gradient in under-5 mortality in Myanmar, which remained after adjusting for the individual proximate determinants. Parents’ education was associated with child mortality before the age of five in the univariate analysis and also after adjusting for the proximate determinants.

Moreover, our study also provides estimates of the association between parents’ socioeconomic status and early childhood mortality within different age groups. Household wealth was associated with mortality to a larger extent during the post-neonatal period after adjusting for the individual proximate determinants. Higher parental education was associated with lower mortality in children aged between one and five years after adjusting for the individual proximate determinants. Benefits from higher parental education were not observed in the neonatal period.

The models (B) that assessed associations between each of the independent variables and the childhood mortality outcomes in the presence of the proximate determinants (birth interval, birth order, mothers’ use of cigarettes/tobacco, maternal age at birth and sex of the child) were preferred for statistical and conceptual reasons. Goodness of fit was assessed and the estimates were less likely to be biased compared with models that additionally adjusted for distal determinants and nutrition and postnatal care since these variables could be part of the causal pathway.

The risk of childhood mortality within all age groups was lowest in the richest families, and in families with post-secondary parental education. These findings are in line with previous studies suggesting that poorer and less educated groups have higher childhood mortality in LMICs [12]. Although under-5 mortality was also attributed to other distal socioeconomic determinants in the multiple logistic models, wealth remained independently protective within the richest households. This is broadly consistent with previous studies conducted in South Africa [28], Kenya [29], Tanzania [30] and Iran [31] which showed that higher socioeconomic status was protective of early childhood mortality. For instance, the results of population surveys conducted in Tanzania [30] and Iran [31] showed that, for under-5 and infant mortality, respectively, children born to the poorest families were more than twice as likely to die compared to those born to the wealthiest families.

With respect to parental education, many studies report that parental education is associated with the families’ socioeconomic circumstances and that parents’ health knowledge and behaviours can impact on child health and survival [15,32–43]. A recent study that used DHS data from 43 LMICs, reported that the role of parental education in child health has attenuated considerably over time (1991 to 2016) in low resource settings and that the association between mother’s education and child health is weakening in line with societal changes [17]. However, our study found that parental education has protective effects on childhood mortality suggesting the parental education remains important in relation to childhood mortality in Myanmar. The findings also reinforce other evidence of protective effects of parental education on childhood mortality in Tanzania [30], India [33] and Palestine [44].

In our study the crude association between wealth and childhood mortality was more evident between one month and five years of age than in the neonatal period. Association between parental education and childhood mortality was not evident during the neonatal period after adjusting for proximate determinants. However, this does not imply that parental education was not indirectly associated with neonatal mortality. The major causes of neonatal deaths in Myanmar are preterm birth, birth asphyxia, neonatal jaundice and congenital abnormalities [45]. The precise aetiology of these major causes together with, or independent of, possible confounding and mediating effects by socioeconomic and other factors is not yet fully understood [46–50]. This could be one reason why we did not observe significant association between the socioeconomic independent variables and mortality during the first 28 days of life. On the other hand underlying causes of deaths after the neonatal period are mainly due to conditions such as pneumonia and diarrhoea, and malnutrition, all factors which can be explained by socioeconomic and environmental conditions [14,45,51].

### Strengths and limitations

We acknowledge that there are several limitations. Since data on birth histories and child mortality were collected retrospectively in the DHS, the quality of this information was dependent on the ability of the mothers to recall their birth histories. We assumed that all mothers were able to recall the death of their child; however, it is possible that there were inaccuracies when reporting age at death and date of death [21]. In addition, the socioeconomic characteristics of the respondents measured at the time of survey may not reflect the characteristics at the time of the child’s mortality although this was assumed in conducting the analysis of association between socioeconomic factors and the mortality outcomes. For instance, the household wealth status of the parents at the time of the survey may have been different to that at the time of the child’s death.

It was not possible to measure all the proximate determinants suggested by Mosley and Chen. For example, specific data on environmental contamination was not available, although information on the source of household water and type of sanitation was included in the calculation of the household wealth index [21]. Moreover, data on injury was not available. It was also not possible to explicitly measure the nutritional status of the children or personal illness control factors, although they are acknowledged as important proximate determinants of early childhood survival.

We further acknowledge that the two variables used to indicate ‘breastfeeding’ and ‘postnatal care within two months’ did not explicitly capture nutritional status and health seeking behaviour. Further, the questions referring to breastfeeding and postnatal care referred to only the mothers’ latest birth. It is also possible that some other variables may have acted either as confounders, mediators or both. For instance, distal socioeconomic factors may have mediated and/or confounded associations between the two independent variables and the mortality outcomes.

Despite these limitations, estimates of levels and trends in childhood mortality provided by DHS are generally reliable [12,17,52,53]. The DHS is the main source of data for childhood mortality in LMICs where vital registration systems are often inadequate [12]. In addition, MDHS is a nationally representative survey, and sample weights were included within all the statistical models to ensure that the results were as representative of the Myanmar population as possible. Moreover, the DHS is a rich data source which allowed us to incorporate Mosely and Chen’s analytical framework to investigate a number of determinants of inequalities in early childhood mortality in Myanmar.

### Conclusions

The findings add to knowledge about associations between socioeconomic indicators and age-specific early childhood mortality in Myanmar. This study provides much needed empirical data that can be used to inform policies to address socioeconomic inequalities in early childhood mortality. Further studies are needed to build upon the evidence base. Policies aimed at reducing early childhood mortality in Myanmar must target socioeconomic disparities. Strategies and interventions that focus on alleviating poverty and improving education need to be adopted within broader policy frameworks.

## Paper context

This study identifies nuanced age-related differences in associations between household wealth and parental education, and early childhood survival before five years of age in Myanmar. It is essential that strategies and interventions aimed at reducing early childhood mortality address socioeconomic determinants such as household wealth and parental education within broader policy frameworks.
